# Identification of Differentially Expressed Human Endogenous Retrovirus Families in Human Leukemia and Lymphoma Cell Lines and Stem Cells

**DOI:** 10.3389/fonc.2021.637981

**Published:** 2021-04-29

**Authors:** Kristina Engel, Lisa Wieland, Anna Krüger, Ines Volkmer, Holger Cynis, Alexander Emmer, Martin S. Staege

**Affiliations:** ^1^ Department of Surgical and Conservative Pediatrics and Adolescent Medicine, Martin Luther University Halle-Wittenberg, Halle, Germany; ^2^ Department of Neurology, Martin Luther University Halle-Wittenberg, Halle, Germany; ^3^ Department of Drug Design and Target Validation, Fraunhofer Institute for Cell Therapy and Immunology, Halle, Germany

**Keywords:** gene expression, RNA sequencing, leukemia, lymphoma, hematopoietic stem cells (HSCs), embryonic stem cells (ESCs), human endogenous retroviruses (HERVs), LL-100 panel

## Abstract

Endogenous retroviruses (ERVs) are becoming more and more relevant in cancer research and might be potential targets. The oncogenic potential of human ERVs (HERVs) has been recognized and includes immunosuppression, cell fusion, antigenicity of viral proteins, and regulation of neighboring genes. To decipher the role of HERVs in human cancers, we used a bioinformatics approach and analyzed RNA sequencing data from the LL-100 panel, covering 22 entities of hematopoietic neoplasias including T cell, B cell and myeloid malignancies. We compared HERV expression in this panel with hematopoietic stem cells (HSCs), embryonic stem cells (ESCs) and normal blood cells. RNA sequencing data were mapped against a comprehensive synthetic viral metagenome with 116 HERV sequences from 14 different HERV families. Of these, 13 HERV families and elements were differently expressed in malignant hematopoietic cells and stem cells. We found transcriptional upregulation of HERVE family in acute megakaryocytic and erythroid leukemia and of HERVFc family in multiple myeloma/plasma cell leukemia (PCL). The HERVFc member HERVFc-1 was found transcriptionally active in the multiple myeloma cell line OPM-2 and also in the Hodgkin lymphoma cell line L-428. The expression of HERVFc-1 in L-428 cells was validated by qRT-PCR. We also confirm transcriptional downregulation of ERV3 in acute megakaryocytic and erythroid leukemia, and HERVK in acute monocytic and myelocytic leukemia and a depression of HERVF in all malignant entities. Most of the higher expressed HERV families could be detected in stem cells including HERVK (HML-2), HERV-like, HERVV, HERVT, ERV9, HERVW, HERVF, HERVMER, ERV3, HERVH and HERVPABLB.

## Introduction

Retroviruses (Retroviridae family) are characterized by a replication cycle in which the viral RNA genome is reverse-transcribed and integrated into a host cell’s nuclear genome to form the provirus. The integration of retroviruses into germline DNA can lead to the formation of vertically transmittable proviral sequences known as endogenous retroviruses (ERVs). Such proviruses maintain the potential to generate multiple germ line copies, either by infectious cycles or retrotransposition (1, 2). Vertebrate genomes typically contain thousands of ERV loci. As a result, the human genome contains approximately 8% HERV sequences (3). In comparison, only 1-2% of the genome codes for essential proteins (4). Based on sequence similarities ERVs have been classified into three major groups. Class I ERVs are related to gammaretroviruses (homology with the Moloney murine leukemia virus MoMuLV) and include the human ERV families HERVE, HERVF, HERVT, HERVV, ERV3 (HERVR), ERV9, HERVW, HERVFRD, HERVH, HERVFc, HERVMER and HERVPABLB. Class II ERVs are related to betaretroviruses and include the human HERVK family and the mouse mammary tumor virus, Class III ERVs are related to spumaretroviruses and include the HERVL family (5–10). In total, ERVs contain more than 200 different groups and subgroups, and so far no uniform classification and nomenclature of ERVs has been used (11).

Retroviral proviruses typically have the three main coding regions *gag* (group-specific antigen), *pol* (transcriptase/polymerase) and *env* (envelope), which are flanked by long terminal repeat sequences (LTRs). Most of this viral DNA is inactivated by deletions or by mutations that have led to the disruption of open reading frames (ORF). Nevertheless, there are a few HERVs with complete and intact open reading frames for the generation of viral proteins (1). Among the HERV families, the HERVK group includes the highest number of members that still have complete sequences for viral genes (12, 13).

The presence of gag, pol and env proteins in the human organism, especially under pathological conditions, has been demonstrated in several studies (14–18). The envelope protein of HERVK inhibits the proliferation of human immune cells, regulates the expression of numerous cytokines and is an example of the control of gene expression by an HERV protein (19). Besides the induction of immunosuppression, HERV proteins can cause cell fusion (20, 21) and might act as superantigens for T cell stimulation (22, 23).

In addition to direct effects of HERV products, HERV-related sequences in the genome can have the ability to control genes in coordinated networks of transcriptional regulation. HERV can act as cis-regulatory elements (24, 25), produce non-coding RNAs that influence nearby genes and/or the global transcriptome in trans (26, 27), and alternate the epigenetic landscape in cancer cells (28, 29). For example, HERVK and HERVH have been shown to influence the transcription of genes involved in pluripotency (30, 31).

The HERV expression is upregulated in various types of cancer like germ cell tumors (32), teratocarcinoma (33), breast cancer (34), prostate cancer (17), Hodgkin lymphoma (35), sarcoma (36) and melanomas (37). Systematic investigations of gene expression data for expressed HERVs and other repetitive elements in cancer revealed a strong impact of these elements on anti-cancer immunity and immunotherapy (38–42).

In summary, the disease causing potential of HERVs is widely accepted. This underlines the importance of HERVs for disease and health and the growing interest in their biology and role in the oncogenesis in different entities. Several studies showed the expression of HERVs in individual cell lines of leukemia and lymphoma and discussed a possible role of endogenous retroviral elements in the development of these cancers. Recently, RNA sequencing (RNA-seq) based gene expression data from the LL-100 panel of leukemia and lymphoma cell lines were made available, which covers 22 different entities including T cell, B cell and myeloid malignancies and thus the entire spectrum of these diseases. This dataset includes cell lines from the following entities: acute myeloid leukemia (AML) with myelocytic, monocytic, erythroid, and megakaryocytic differentiation; chronic myeloid leukemia (CML) with myeloid or lymphoid blast crises; multiple myeloma/plasma cell leukemia (PCL); T cell and pre B cell acute lymphoblastic leukemia (ALL); Hodgkin lymphoma; Burkitt lymophoma/B cell ALL; chronic lymphocytic leukemia/prolymphocytic leukemia (CLL/PLL); activated B cell (ABC) and germinal center (GC) subtypes of diffuse large B cell lymphoma (DLBCL); hairy cell leukemia (HCL); mantle cell lymphoma (MCL); primary effusion lymphoma (PEL); primary mediastinal B cell lymphoma (PMBL); anaplastic large cell lymphoma (ALCL). The panel is completed by cell lines from myeloproliferative neoplasm (MPN), NK cell and mature T cell malignancies. The LL-100 panel consists of well-characterized and authenticated cell lines that are publicly available (www.dsmz.de) and widely used in many laboratories (43). In the present investigation, we used this comprehensive RNA-seq data collection in order to identify differentially expressed HERV families in hematopoietic cancer cells in comparison with hematopoietic stem cells (HSCs) and embryonic stem cells (ESCs). The aim of this investigation was to characterize HERV expression in this widely used cell lines panel and to identify HERVs that are expressed in individual entities. These HERVs might represent candidates for future investigations using larger numbers of patient samples.

## Material and Methods

### Construction of a Synthetic Virus Metagenome

To quantify HERV expression, a synthetic virus metagenome was assembled to be used for mapping analysis. Because endogenous viral sequences can influence the development of cancer and immune diseases by transcription of *gag*, *pol* and *env* genes as well as by synthesis of viral proteins, publications and databases were searched for HERVs with full-length sequences and/or open reading frames. A total of 116 individual human endogenous viral genomic sequences and 3 sequences of endogenous bornavirus-like nucleoprotein (EBLN) elements were collected from the nucleotide database from the National Center for Biotechnology Information (NCBI). The corresponding reference publications and accession numbers are listed in the **Supplementary Table 1**. These HERV sequences belong to 14 HERV families and were assembled to a synthetic virus metagenome. Additionally, 124 sequences of exogenous viruses and non-human endogenous viruses and 4 sequences of housekeeping genes were integrated as spacers. To assemble the synthetic virus metagenome, the sequences of all endogenous retroviruses and exogenous viruses were copied one after the other in a text file. The sequence order corresponds to the order in **Supplementary Table 1**. Using the biological sequence alignment editor BioEdit 7.2 (https://bioedit.software.informer.com), the created sequence was saved as a fasta file, which was then used as the HERV reference genome for mapping. We have added this fasta file named Viruses21.fasta in the supplement.

### RNA-Sequencing Data Analysis of the LL-100 Panel Dataset and Calculation of the HERV Family Specific FPKMs

The cell lines of the LL-100 panel are authenticated and free of contamination by mycoplasma or non-inherent viruses. Furthermore, the method of RNA isolation and sequencing are identical in all cell lines. Therefore, this dataset allows comparative studies without methodical impact. The RNA-seq data were downloaded from the NCBI Short Read Archive (SRA) under BioProject PRJEB30312 (43).

To analyze the sequencing data they were uploaded to the Galaxy platform (44). With FastQC (Galaxy Version 0.72+galaxy1) we checked the quality of raw sequence data. The paired-end reads were mapped to the synthetic virus metagenome using Bowtie2 (Galaxy Version 2.3.4.3+galaxy0) with default settings (45). The overall alignment rate of the LL-100 cell lines varied between 0.44 and 1.14%. For quantification of gene expression from the BAM files, the mapped reads were counted using FeatureCounts [Galaxy Version 1.6.4+galaxy2 (46)]. The GTF file used for quantification (Viruses21.gtf) is provided in the supplement. Only reads that mapped uniquely to the virus metagenome were counted using the following settings: counted fragments (-p), only allow fragments with both reads aligned (-B), disabled multi-mapping. The multi-mapping option in featureCounts (-M) was not used because this might lead to overestimations (47). However, this is only relevant if the –a/-k option is used for mapping. We used Bowtie2 with the default values from the Galaxy server. Therefore, single reads are mapped only to single positions. This has the advantage that family specific FPKM can be calculated without bias from the family size. For the quantification of the counted fragments with FeatureCounts the Fragments Per Kilo Base per Million (FPKM) values were calculated to account for variation in provirus lengths and total number of reads. To calculate the HERV family specific FPKMs, the mapped fragments for each HERV family member and the length of each HERV family member were summed up and calculated using the following formula: family FPKM = sum of family fragments/sum of gene lengths of all family members *1,000/total number of reads*1,000,000. For differential expression analysis the HERV family specific FPKMs were compared with the FPKMs in PBMCs as a universal control. Calculation of FPKM and family specific FPKM was performed in Micosoft Excel 2016 (see Supplement). In addition, HERV family specific expression normalized to reference genes was calculated. The three housekeeping genes hypoxanthine phosphoribosyltransferase 1 (*HPRT1*), glyceraldehyde-3-phosphate dehydrogenase (*GAPDH*) and ubiquitin B (*UBB*) were used. Analogous to the HERV family specific FPKM, the housekeeping gene specific FPKM was calculated and HERV family specific FPKM were normalized to this value (see Supplement).

### RNA-Sequencing Data Analysis of Datasets From Stem Cells and Normal Blood Cells

Three paired-end RNA-seq datasets (SRR2453342, SRR2453343, SRR2453346) from human pluripotent embryonic stem cells (ESCs) from the BioProject PRJNA296379 were analyzed (48). The reads were mapped against the virus metagenome analogous to the LL-100 RNA-seq data. From the BioProject PRJNA437152 (49) three paired-end RNA-seq datasets (SRR6811702, SRR6811703, SRR6811704) of fetal liver hematopoietic stem and progenitor cells (HSCs) were used. In addition, two RNA-seq datasets (SRR6298381, SRR6298352) from normal peripheral blood mononuclear cells (PBMCs) from the BioProject PRJNA418779 (50) were used. These RNA-seq data from stem cells and normal blood cells were mapped against the virus metagenome analogous to the LL-100 RNA-seq data. The overall alignment rate was as follows: for ESCs 1.78 - 2.50%, for HSCs 0.13 - 0.16% and for PBMCs 0.68 - 0.75%.

### Cell Lines and Culture Conditions

The five Hodgkin lymphoma cell lines L-1236, L-428, L-540, KM-H2 and HDLM-2 were obtained from the German Collection of Microorganisms and Cell Cultures GmbH (Braunschweig, Germany). Cell lines were grown in 5% CO_2_ atmosphere in a humidified 37°C incubator and cultured in RPMI (Pasching, Germany), supplemented with 10% (v/v) fetal calf serum (FCS), 100 U/mL penicillin and 100 μg/mL streptomycin (Life Technologies, Carlsbad, CA, USA). The cells were passaged in 75cm^2^ flask twice a week.

### Preparation of RNA Samples

Total RNA was extracted using the NucleoSpin RNA Mini kit for RNA purification (Macherey-Nagel GmbH & Co. KG, Düren, Germany) according to manufacturer’s instructions. Quality and concentration of RNA were determined by visualization of rRNA bands in agarose gel electrophoresis and with a NanoDrop 2000 spectrophotometer (Thermo Fisher Scientific, Waltham, MA, USA).

### Quantitative Reverse Transcription-Polymerase Chain Reaction (qRT-PCR)

To quantify HERVFc-1 transcripts, 2 μg of total RNA and 100 μM Oligo-dT primer were used for cDNA synthesis (M-MLV Reverse Transcriptase, RNase H Minus-Kit; Promega GmbH, Walldorf, Germany) following the manufacturer’s instructions. A sample without reverse transcription was also prepared to check for genomic DNA contamination (noRT control).

For the qRT-PCR 5 μL 5xGreen GoTaq buffer, 0.2 μl GoTaq polymerase (both Promega GmbH, Mannheim, Germany), 16.8 μl nuclease-free water, 0.5 μl of 10 mM dNTPs (Thermo Fisher Scientific, Waltham, MA, USA), 0.25 μl forward primer (25 μM), 0.25 μl reverse primer (25 μM) and 2 μl cDNA were prepared. The amplification was performed under the following conditions: 94°C for 30 s, 60°C for 30 s, 72°C for 45 s (40 cycles). The experiment was repeated four times for the gene expression analysis.

Relative gene expression was calculated with the 2^-ΔΔCt^ method (51) using hypoxanthine phosphoribosyltransferase 1 (*HPRT1*) as reference gene for normalization. noRT controls were used for exclusion of genomic DNA amplifications. The following primers from Eurofins Genomics GmbH (Ebersberg/Germany) were used: HERVFc-1_forward: 5’-CTC CCC ATC TCT CTG GTG C-3’ and HERVFc-1_reverse: 5’-TGA GGA GGC TGG TTT CTC TAA G-3’; HPRT1 _forward: 5’-ACC AGT CAA CAG GGG ACA TAA-3’ and HPRT1_reverse: 5’-CTT CGT GGG GTC CTT TTC ACC-3’.

### Data Visualization and Statistical Analysis

Data visualization and One-way ANOVA followed by Dunnett’s multiple comparisons test for statistical analysis was performed using GraphPad Prism version 8.0.0 for Windows, GraphPad Software, San Diego, CA, USA. Statistical significances are symbolized as asterisks: * = p < 0.05, ** = p < 0.01, *** = p < 0.001, **** = p < 0.0001).

## Results

We analyzed the RNA-seq data of 100 cell lines from the LL-100 panel covering 22 entities of human leukemia and lymphoma (43). Eight RNA-seq data from PBMCs and HSCs and ESCs were also examined for comparison. Our investigation included 116 sequences of 14 HERV families and 3 sequences of the EBLN family.

### Detection of EBV, HHV8 and XMRV in Cell Lines of LL-100 Panel

**Figure 1:**
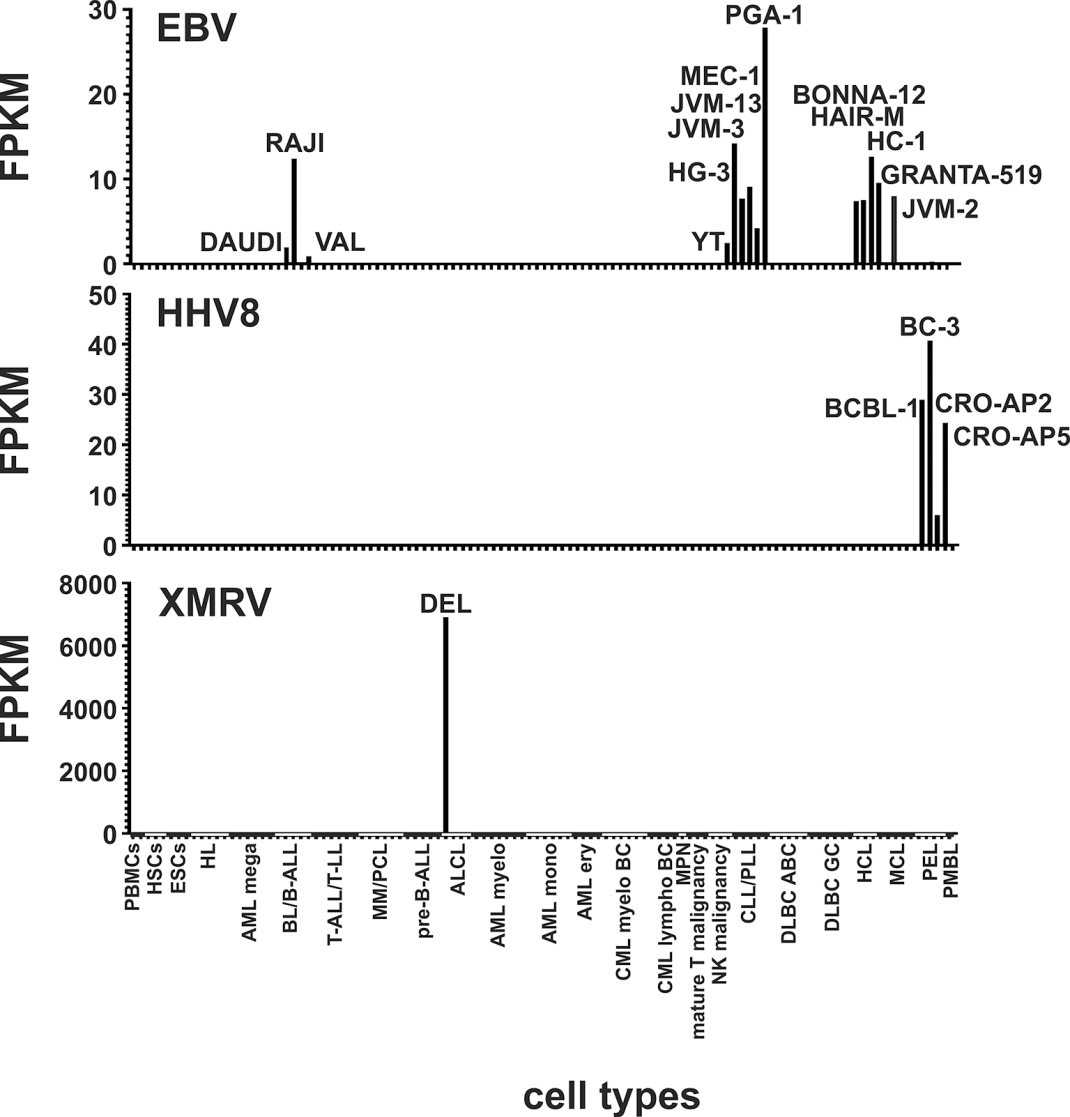
Detection of EBV, HHV8 and XMRV in leukemia and lymphoma cell lines and stem cells. Mapped reads against the EBV gene *EBNA-1*, the HHV8 gene *ORF57* and against the XMRV genome were counted and FPKMs calculated. Raw data are available in **Supplementary Table 2**.

Some cell lines of the LL-100 panel are known to contain Epstein-Barr virus (EBV), Human herpes virus 8 (HHV8) or xenotropic MuLV-related virus (XMRV). To demonstrate the functionality of our method we first analyzed all cell lines for the presence of these exogenous viruses. As expected, we could detect reads mapping against the EBV gene *EBNA-1* in the EBV-positive cell lines DAUDI, RAJI, VAL, YT, HG-3, JVM-3, JVM-13, MEC-1, PGA-1, BONNA-12, HAIR-M, HC-1, GRANTA-519 and JVM-2 (**Figure 1**, **Supplementary Table 2**). *EBNA-1* is the only latent protein-encoding gene identified that is expressed consistently in Burkitt’s lymphoma cells with EBV latency type I. It is believed to contribute to EBV malignancies by B cell-directed expression (52). In the HHV8-positive primary effusion lymphoma (PEL) cell lines BC-3, BCBL-1, CRO-AP2, CRO-AP5 we also verified the presence of this virus by detection of *ORF57* specific reads. The *ORF57* gene product is essential for lytic HHV8 replication and virion production (53). PEL cases are universally associated with Kaposi sarcoma herpesvirus/HHV8 (54). XMRV was detectable only in the single XMRV-positive cell line DEL (**Figure 1**, **Supplementary Table 2**).

### Differential Expression of HERVE, ERV3 and HERVK in Leukemia Cell Lines

**Figure 2:**
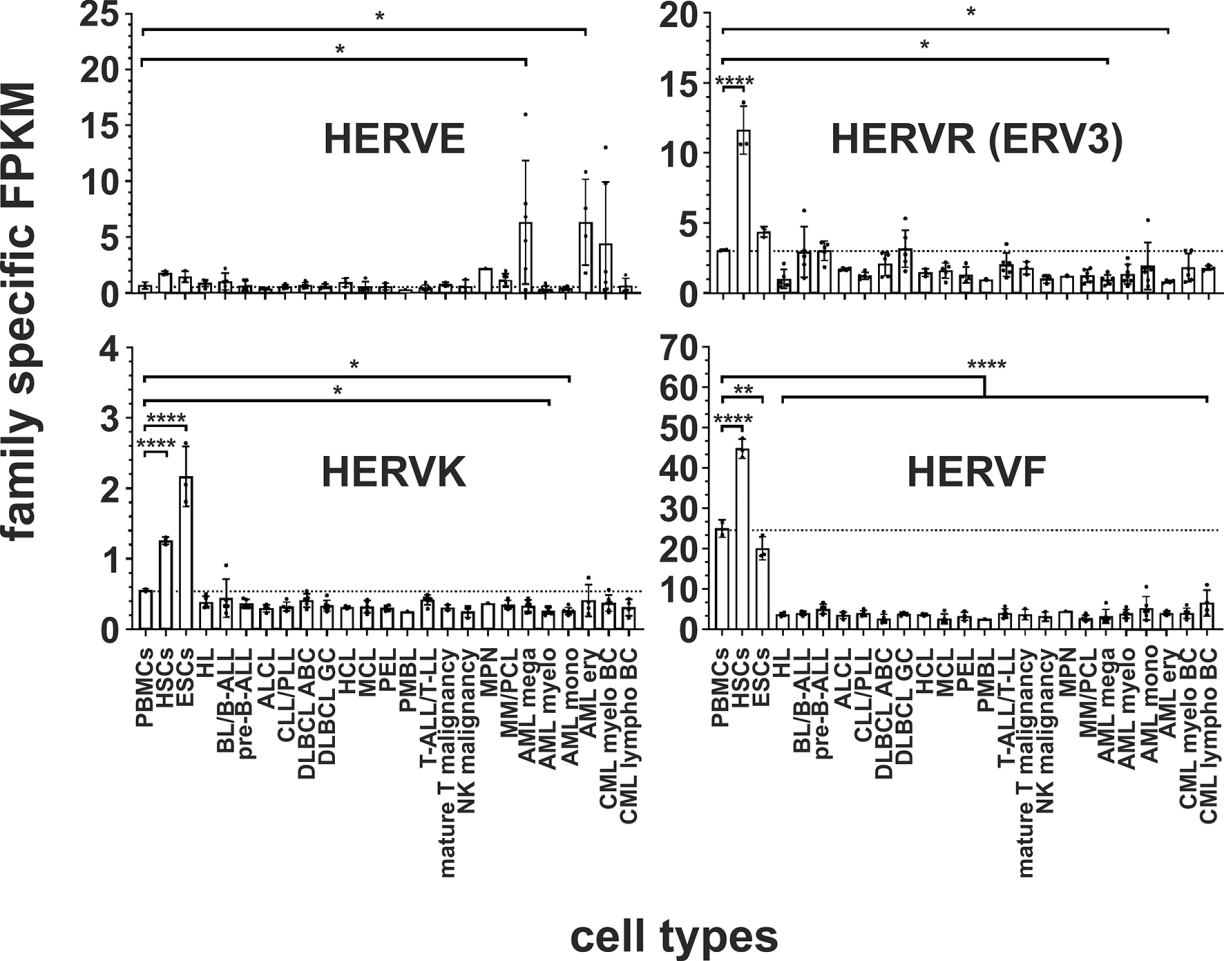
Differential expression of HERV families HERVE, ERV3, HERVK and HERVF in leukemia and lymphoma cell lines and stem cells. For each cancer entity and stem cell type, family specific FPKMs were calculated. Black dots marked RNA-seq data from individual cell lines or stem cell samples. The bar graphs represent means and error bars indicate standard deviations. For statistical analysis, the mean values of the individual entities and stem cells were used for multiple comparisons. The dotted line represents expression in PBMCs. Raw data are available in **Supplementary Table 3**. p-value: * = p < 0.05, ** = p < 0.01, **** = p < 0.0001.

For our mapping analysis we have added only almost complete HERV sequences including *gag*, *pol* and *env* genes to our virus metagenome. We found only one known full-length HERVE sequence known as ERVE-1 in the human NCBI nucleotide database. Within the human genome there is a large number of HERVE related sequences that are 90-93% identical to the known full-length ERVE-1 sequence (55). Although only the ERVE-1 sequence is present in the virus metagenome, the short length of the 100-150 bp reads and the high homology of the HERVE sequences should also allow the filtering and quantification of HERVE related reads if they are transcriptionally active in human cells. A significant transcriptional upregulation for HERVE (p < 0.05) was identified in megakaryocytic AML cell lines (CMK, ELF-153, M-07e, MEGAL, MKPL-1, UT-7) and in erythroid AML cell lines (F-36P, HEL, OCI-M2, TF-1), but not in myelocytic AML and monocytic AML cell lines or in HSCs and lymphoma cells (**Figure 2**).

The human genome contains about 40 ERV3-like elements (56–58). However, only the ERV3-1 copy has a complete ORF for an env protein (56) and is included in our virus metagenome. No significant transcriptional overexpression of ERV3 was found in the analyzed leukemia and lymphoma entities. However, compared to PBMCs, significantly less ERV3 specific reads were detected in cell lines for the two AML entities megakaryocytic AML and erythroid AML (p < 0.05, **Figure 2**).

The human genome contains around 100 integrated copies of the HERVK (HML-2) virus (47), of which we have included 92 different full-length HERVK sequences for analysis to cover a complete spectrum of the HERVK (HML-2) family (11, **Supplementary Table 1**). Significantly reduced transcripts (p < 0.05) were identified for HERVK in the two AML entities myelocytic AML (cell lines EOL-1, HL-60, KASUMI-1, KG-1, NB-4, OCI-AML3, SKNO-1) and monocytic AML (cell lines ME-1, MOLM-13, MONO-MAC-6, MUTZ-3, THP-1, U-937) (**Figure 2** and **Supplementary Table 3**). For the HERVFRD and EBLN families no significant differential expression was obtained in malignant cells (**Supplementary Figure 1**).

### Transcriptional Downregulation of HERVF in Leukemia, Lymphoma and Malignant Diseases

The endogenous retrovirus group 48 member 1 (ERVH48-1/ERV-Fb) is part of the HERVF family, whose family members are preferentially expressed in the human placenta (59). Our data show that, compared to PBMCs, the transcription of this HERVF element is even downregulated in all of the leukemia and lymphoma cell lines (p < 0.0001, **Figure 2**).

### Transcriptional Upregulation of HERV Families in HSCs and ESCs

The activation of endogenous retroviral LTRs plays a fundamental role in the maintenance of pluripotency and induction of an antiviral state in stem cells (60). Therefore, we have compared the differential expression of the 14 HERV families and elements in PBMCs and leukemia and lymphoma cell lines with human pluripotent ESCs cells (n=3) and human HSCs (n=3).

**Figure 3:**
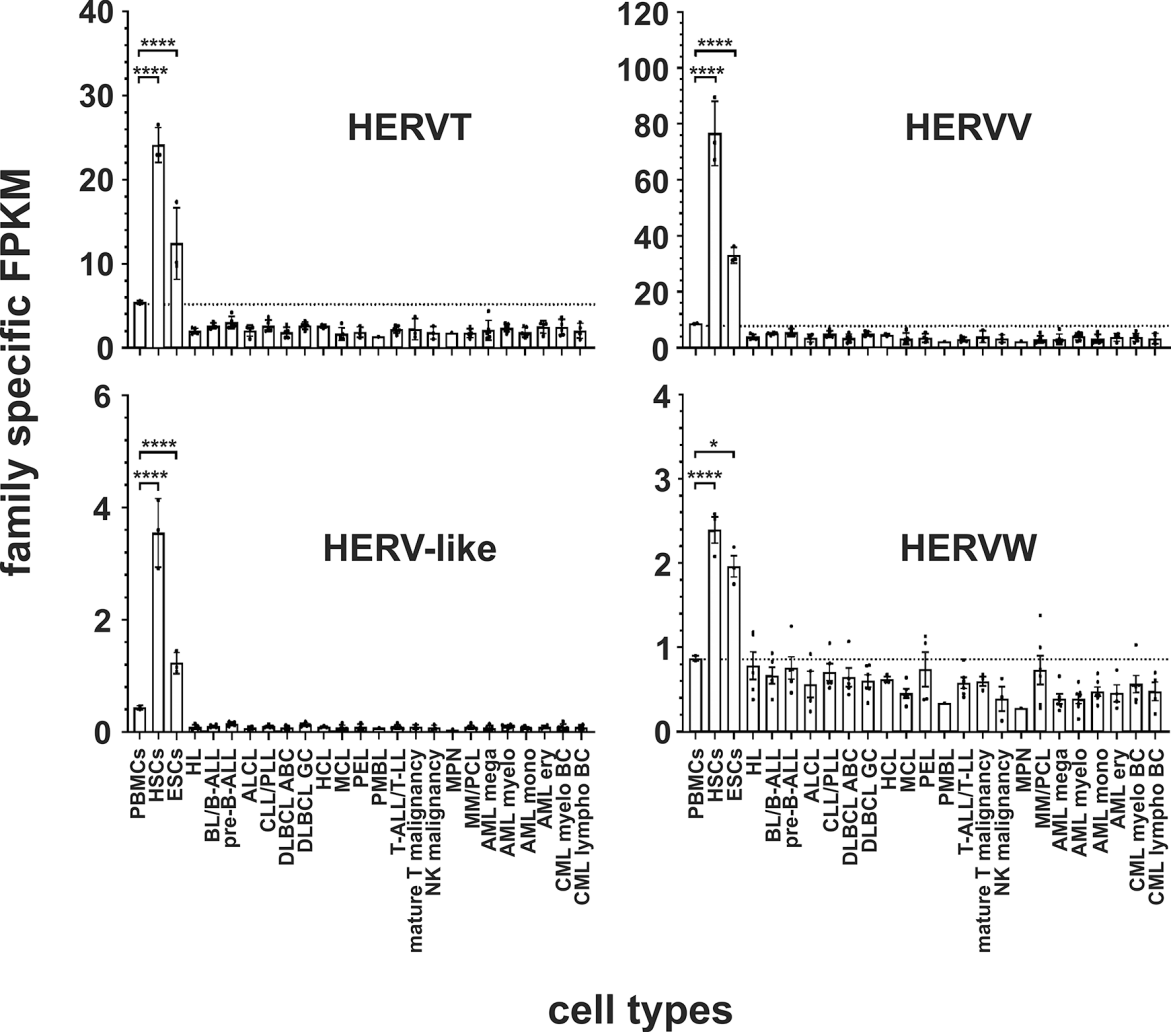
Differential expression of HERV families HERVT, HERVV, HERV-like and HERVW in leukemia and lymphoma cell lines and stem cells. For each cancer entity and stem cell type, family specific FPKMs were calculated. Black dots marked RNA-seq data from individual cell lines or stem cell samples. The bar graphs represent means and error bars indicate standard deviations. For statistical analysis, the mean values of the individual entities and stem cells were used for multiple comparisons. The dotted line represents expression in PBMCs. Raw data are available in **Supplementary Table 3**. p-value: * = p < 0.05, **** = p < 0.0001.

**Figure 4:**
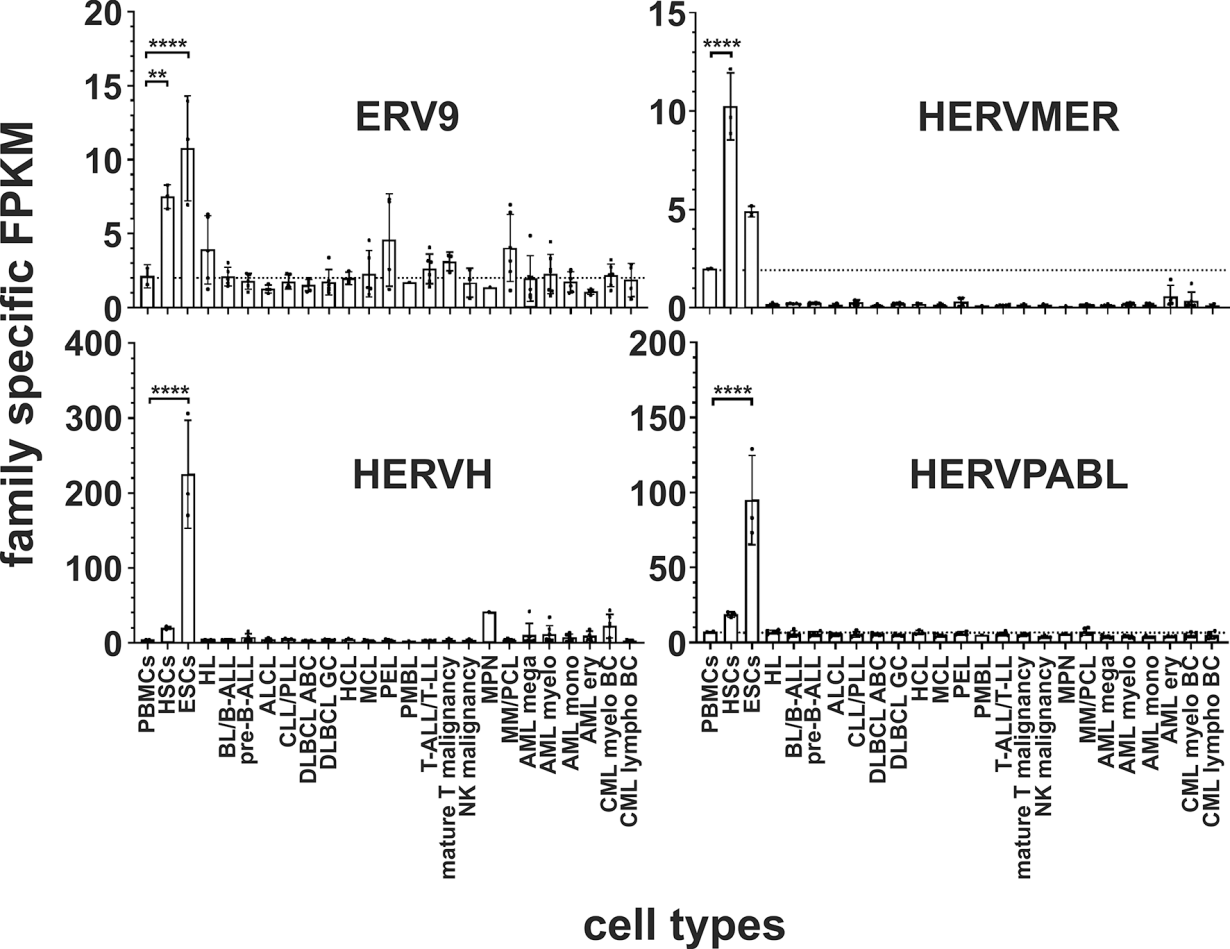
Differential expression of HERV families ERV9, HERVMER, HERVH and HERVPABLB in in leukemia and lymphoma cell lines and stem cells. For each cancer entity and stem cell type, family specific FPKMs were calculated. Black dots marked RNA-seq data from individual cell lines or stem cell samples. The bar graphs represent means and error bars indicate standard deviations. For statistical analysis, the mean values of the individual entities and stem cells were used for multiple comparisons. The dotted line represents expression in PBMCs. Raw data are available in **Supplementary Table 3**. p-value: ** = p < 0.01, **** = p < 0.0001.

In both stem cell types, nine HERV families and elements are each significantly upregulated compared to PBMCs. The HERVK family, HERV-like family, HERVV family, HERVT family were all highly significantly upregulated (all p < 0.0001) in both stem cell types (**Figures 2** and **3**). The higher expression of ERV9 was more significant in ESCs (p < 0.0001) compared to HSCs (p < 0.01, **Figure 4**), whereas the upregulation of HERVW and HERVF was more pronounced in HSCs (both p < 0.0001) than in ESCs (HERVW: p < 0.05, HERVF: p < 0.01; **Figures 2** and **3**). HERVMER and ERV3 were only significantly differentially expressed (p < 0.0001) in HSCs, while HERVH and HERVPABLB were significantly upregulated (p < 0.0001) only in ESCs (**Figures 2** and **4**). No significant differential expression in HSCs or ESCs was observed for the HERVFRD family and the EBLN family (**Supplementary Figure 1**).

### Transcriptional Upregulation of HERVFc Family in Multiple Myeloma/PCL Cell Lines

**Figure 5:**
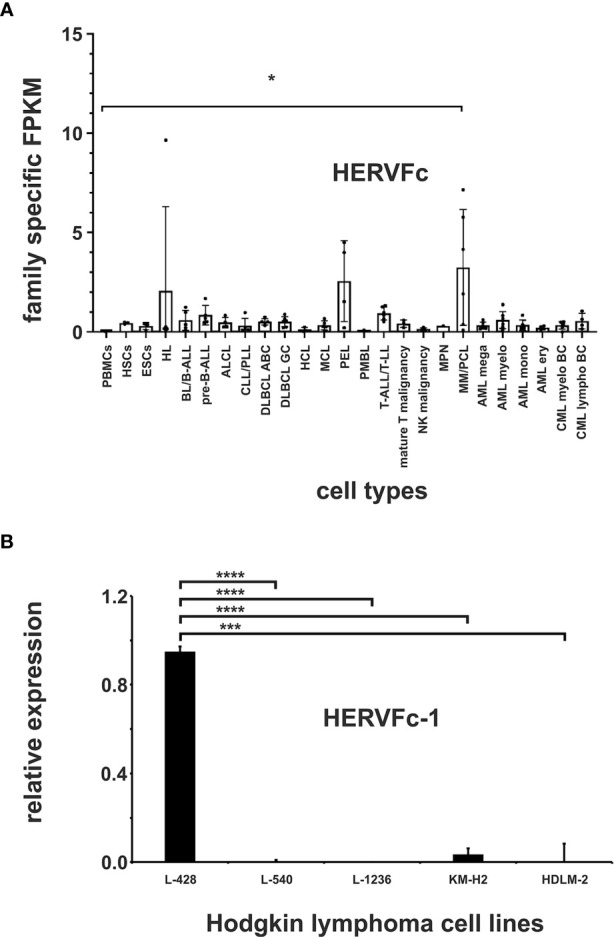
Expression analysis of the HERVFc family. **(A)** Differential expression of HERV family HERVFc in leukemia and lymphoma cell lines and stem cells. For each cancer entity and stem cell type, family specific FPKMs were calculated. Black dots marked RNA-seq data from individual cell lines or stem cell samples. The bar graphs represent means and error bars indicate standard deviations. For statistical analysis, the mean values of the individual entities and stem cells were used for multiple comparisons. p-value: * = p < 0.05. Raw data are available in **Supplementary Table 3**. **(B)** qRT-PCR analysis of HERVFc-1 expression in Hodgkin lymphoma cell lines. For the comparative analysis, *HPRT1* was used as reference. The bar charts correspond to the mean of four independent experiments. The error bars indicate the standard deviation. p-value: *** = p < 0.001, **** = p < 0.0001.

The HERVFc subfamily is part of the larger HERVF family (61). The human genome comprises only six known HERVFc family members, among which two possess full-length coding envelope genes (62). Therefore HERVFc-1 (Fc1env, Xq21.33) and HERVFc-2 (envF(c)2, 7q36.2) were added to the virus metagenome. A significantly increased transcription of HERVFc family specific transcripts (p < 0.05) was only detected in cell lines from multiple myeloma/plasma cell leukemia (PCL) (cell lines KMS-12-BM, L-363, LP-1, OPM-2, RPMI-8226 and U-266; **Figure 5A**; **Supplementary Table 3**). The cell lines from Hodgkin lymphoma and from primary effusion lymphoma (PEL) show at least a tendency for higher expression of HERVFc. Transcriptional upregulation is mainly limited to HERVFc-2, which is poorly expressed in other lymphoma and leukemia cell lines and stem cells (**Figure 5A** and **Supplementary Table 3**). HERVFc-1 -specific reads could only be detected in two cell lines from different diseases including Hodgkin lymphoma cell line L-428 (23 fragments = 0.08 FPKM) and multiple myeloma/PCL cell line OPM-2 (51 fragments = 0.2 FPKM); see **Supplementary Table 3**.

### Transcriptional Expression of HERVFc-1 in the Hodgkin Lymphoma Cell Line L-428

To confirm the results obtained in the mapping analyses and to further verify the functionality of our mapping approach, the expression of HERVFc-1 in the Hodgkin lymphoma cell lines L-428, L-540, L-1236, KM-H2 and HDLM-2 was analyzed by RT-PCR. According to the mapping results for HERVFc-1 (**Supplementary Table 3**) high expression of this HERV element was only detectable by RT-PCR in the Hodgkin lymphoma cell line L-428 (p < 0.0001), but not in the cell lines L-540, L-1236, KM-H2 and HDLM-2 (**Figure 5B**). These data confirm the functionality of our mapping approach to identify differentially expressed HERV sequences in RNA-seq data. Normalization of HERV family specific FPKM to housekeeping genes had no major effect on the detectability of entity specific HERV expression (see **Supplementary Table 3**).

## Discussion

Several studies showed the expression of HERVs in individual leukemia and lymphoma cell lines and discussed a possible role of endogenous retroviral elements in the development of these cancers. In this study, we analyzed differentially expressed HERV families in a collection of cell lines from a broad spectrum of hematopoietic entities to demonstrate the association of these transcriptionally regulated HERVs with leukemia and lymphoma. 13 HERV families and elements were found to be expressed differentially in malignant cells and stem cells. While only some HERV families were upregulated or downregulated in certain cancers, most of the higher expressed HERV families could be detected in stem cells (**Figures 2**–**5**).

Our virus metagenome analyses confirm the activation of HERVE in two AML entities: megakaryocytic AML and erythroid AML (**Figure 2**). Expression of ERVE-1 *env* gene, the only full-length HERVE member, was observed in ovarian cancer (63) and prostate cancer (64). The *env* gene is also expressed in many normal human tissues (*e.g.* brain, kidney, testes, placenta, thymus, uterus), suggesting that the HERVE family is expressed according to the transcriptional program of human tissues and human cancer cells (65). For example, the HERVE family is involved in the transcriptional regulation of the genes for apolipoprotein C-I and endothelin B receptors (66) and the human growth factor pleiotrophin in the placenta by contributing alternative promoters (67). Transcripts of HERVE were obtained from cells of the chronic myeloid leukemia cell line K562 and from the T cell leukemia cell line HSB-2 (55). Differences in the transcriptional pattern of HERVE between malignant and non-malignant hematopoietic cells (55) raise questions about the role of these elements in the development of leukemia. HERVE transcription could be activated by inhibition of LTR methylation as shown in lupus erythematosus (68). The relationship between transcriptionally active HERVE elements and adjacent genes in AML needs to be analyzed in further studies. Although the exact function of HERVs in cancer is unclear, the evidence for the involvement of HERVE in megakaryocytic AML and erythroid AML is considerable and further analysis is needed.

In cell lines for the two AML entities, megakaryocytic AML and erythroid AML, significantly less ERV3 specific transcripts are detectable compared to PBMCs (**Figure 2**). From the 40 ERV3-like elements in humans only the ERV3-1 copy on chromosome 7q11.21 has a complete open reading frame for a viral envelope protein (56). ERV3-1 is closely linked to the neighboring ZNF117 locus, but for both genes the physiological functions are not yet understood (69). The role of ERV3-1 in cancer appears to be different in several tumor entities. The overexpression of ERV3-1 is associated with several tumor entities like prostate, lung, liver and colorectal cancer (8, 64, 70). However, in the myelogenous leukemia cell line U-937 ERV3 was found to be upregulated during monocyte differentiation (71) because of demethylation of the ERV3 locus (72). On the other hand, ERV3 was classified as a tumor suppressor, as downregulation of ERV3 has been reported in choriocarcinoma (73, 74). Suppression of ERV3 transcription was also observed in Hodgkin lymphoma cells compared to normal blood cells and growth inhibited Hodgkin lymphoma cells expressed higher levels of ERV3 RNA than proliferating cells (75). Our data also suggest a downregulation of ERV3 in the five HL cell lines analyzed (**Figure 2**). The same was observed for the other lymphoma entities being studied (**Figure 2**). Only in HSCs ERV3 was significantly upregulated. Whether the maintenance of methylation at the ERV3 locus is responsible for downregulation and what is the cause during disease development needs to be investigated in further analyses.

Reduced transcripts were identified also for HERVK in cell lines for the two AML entities myelocytic AML and monocytic AML (**Figure 2**). The HERVK group is the most biologically active class of HERVs (13). The reactivation of HERVK may contribute to the pathogenesis of various diseases, such as ovarian cancer (63), prostate cancer (64), melanoma (76) or multiple sclerosis (77). HERVK has also been found in the plasma of patients with lymphoma (78) and a significantly higher expression of HERVK in acute lymphoblastic leukemia (79), acute myeloid leukemia (80), and chronic lymphocytic leukemia (81), strongly suggests a possible contribution of this HERVs in the pathogenesis of these diseases. We could not confirm an upregulation of HERVK in lymphoma or leukemia cell lines in our analysis. Whether this reflects differences between established cell lines and the situation *in vivo* requires further investigation. In our evaluation we analyzed the summarized expression data of 92 different full-length HERVK sequences. It seems that cancer and immune diseases are not accompanied by transcriptional activation of all HERVs, but rather the upregulation of specific HERV loci in different diseases was observed. Other studies described a more global activation of HERV especially in solid tumor samples (25). Nevertheless, different tumor entities could be classified according to their HERV expression (25) suggesting entity specific expression pattern. Due to the large group of HERVK it cannot be excluded that the differential expression of single loci is not detected by our evaluation. Focusing on single HERVK loci is necessary in a further analysis, also to detect possible deregulated HERVK loci in myelocytic AML, monocytic AML and lymphoma. It can be speculated that specific deregulated HERVK loci are characteristic for these entities.

HERVFc transcription is upregulated in multiple myeloma/PCL (**Figure 5A**). The human genome comprises only six HERVFc family members, among which two possess full-length coding *env* genes. This limited expansion is considered to be evidence for recent integrations in the course of primate evolution. The *env* gene of the HERVFc-1 provirus still codes a full-length protein (62). Upregulation of HERVFc might suggest an involvement in the pathogenesis of multiple myeloma. Differential expression is mainly restricted to HERVFc-2, which is poorly expressed in other lymphoma and leukemia cell lines and stem cells (**Figure 5A** and **Supplementary Table 3**). HERVFc-1 is transcriptionally active only in one multiple myeloma/PCL (OPM-2) cell line and also in the Hodgkin lymphoma cell line L-428 (**Figure 5B** and **Supplementary Table 3**). The HERVFc subfamily is part of the enlarged HERVF family (61). Our data show that expression of HERVF (ERVH48-1) is not associated with multiple myeloma/PCL (**Figure 2**). HERVF is even significantly downregulated in all analyzed leukemia or lymphoma cell lines. This suggests that HERVF might be function as a tumor suppressor.

In human HSCs and ESCs we found several HERV families transcriptionally upregulated. Overexpression of HERVK, HERV-like, HERVV, HERVT, ERV9, HERVW and HERVF family members and elements were found in both. HERVMER and ERV3 are only significant differentially expressed in HSCs, while HERVH and HERVPABLB are significant upregulated only in ESCs (**Figures 2**–**4**). The relevant function of these HERVs appears to be their involvement in the maintenance of cell pluripotency. In further studies, also high levels of HERVK transcripts and proteins are found in undifferentiated ESCs and induced pluripotent stem cells. The expression was inhibited by induction of differentiation (31). The role of HERV LTRs in maintenance of the undifferentiated phenotype and cell proliferation is supported by providing transcription factor-binding platforms for master regulators of pluripotency, such as OCT4, SOX2, and NANOG, which wire the expression of these genes to pluripotency (82). An HERVH driven lncRNAs also influence the transcriptome of the genes involved in pluripotency (30, 83). Also for HERVW an overexpression in pluripotent stem cells was verified (84). Expression of *TP63* and *TNFRSF10B* genes, encoding the p63 homologue of the tumor suppressor p53 and death receptor 5 (DR5), respectively, is regulated by upstream LTRs belonging to the ERV9 group of HERVs, which have an anti-oncogenic effect (85, 86). The importance of ERV9 and the other HERVs like HERVV, HERVT, HERVF, HERVMER, ERV3 and HERVPABLB for the pluripotency of stem cells must be considered in further analyses.

Many studies have shown HERV expression in single cell lines or samples from leukemia and lymphoma patients. One possible reason for our finding of relatively low expression of HERVs in these different tumor cell types can be explained by our approach. We identified HERVs that are specifically transcribed in individual entities and included several entity-specific cell lines. This does not exclude the existence of single cell lines with higher expression. Furthermore, it is necessary to analyze whether there are differences between patient samples and cell lines.

In summary, we provide new insights into the landscape of differentially expressed HERV families in a widely used spectrum of hematopoietic tumor cells and in stem cells. We show that the expression of HERV families is specific for specific entities. The next challenges are related to the characterization of specific HERV elements with respect to their function in gene expression regulation or by expression of viral proteins. Overall, a better understanding of these elements and their role in the development of cancer will provide new applications for the development of new biomarkers for prevention, diagnosis, prognosis and therapy.

## Data Availability Statement

The original contributions presented in the study are included in the article/**Supplementary Material**. Further inquiries can be directed to the corresponding author.

## Author Contributions

KE: data collection, data analysis, interpretation of data, generating figures, and drafting the article. LW: part of data collection, interpretation of data, review, and editing. AK, IV: part of data collection, review, and editing. HC: conception of the work, review, and editing. AE: conception of the work, project administration, review, and editing. MS: conception of the work, supervision, project administration, visualization, and critical revision of the article. All authors contributed to the article and approved the submitted version.

## Funding

The work was supported by grant ZS/2018/12/96228 (MS and AE) and ZS/2018/12/96169 (HC) from European Regional Development Fund within the local program “Sachsen-Anhalt WISSENSCHAFT Schwerpunkte”. We acknowledge the financial support of the Open Access Publication Fund of the Martin Luther University Halle-Wittenberg.

## Conflict of Interest

The authors declare that the research was conducted in the absence of any commercial or financial relationships that could be construed as a potential conflict of interest.

## Abbreviations

ALCL, anaplastic large cell lymphoma; ALL, acute lymphoblastic leukemia; AML, acute myeloid leukemia; BC, blast crisis; BL, Burkitt lymphoma; CLL, chronic lymphocytic leukemia; CML, chronic myeloid leukemia; DLBCL ABC, diffuse large B cell lymphoma, activated B cell subtype; DLBCL GC, diffuse large B cell lymphoma, germinal center subtype; ery, erythroid; ESCs, embryonic stem cells; HCL, hairy cell leukemia; HL, Hodgkin lymphoma; HSCs, hematopoietic stem cells; LL: lymphoblastic lymphoma; mega, megakaryocytic; lympho, lymphocytic; MCL, mantle cell lymphoma; MM: multiple myeloma; mono: monocytic; MPN, myeloproliferative neoplasm; myelo, myelocytic; NK, natural killer; PBMCs, peripheral blood mononuclear cells; PCL, plasma cell leukemia; PEL, primary effusion lymphoma; PLL, prolymphocytic leukemia; PMBL, primary mediastinal B cell lymphoma.
