# Supplementary material for: Identification of Differentially Expressed Human Endogenous Retrovirus Families in Human Leukemia and Lymphoma Cell Lines and Stem Cells
Source: Front Oncol. 2021 Apr 29;11:637981. doi: 10.3389/fonc.2021.637981 (PMC8117144; doi:10.3389/fonc.2021.637981)
Supplement: Supplementary file 1 [file DataSheet_1.zip › 637981_Data_Sheet_1/Engel et al_Supplementary_Material.docx]

Supplementary Material for

Identification of Differentially Expressed Human Endogenous Retrovirus Families in Human Leukemia and Lymphoma Cell Lines and Stem Cells

Kristina Engel, Lisa Wieland, Anna Krüger, Ines Volkmer, Holger Cynis, Alexander Emmer and Martin S. Staege

**This supplement contains:**

- Supplementary figure, table and file legends (this file)
- Supplementary Figure SFig. 1
- Supplementary Table STab. 1
- Supplementary Table STab. 2
- Supplementary Table STab. 3
- Supplementary File Viruses21.fasta
- Supplementary File Viruses21.gtf

**Supplementary Figure legend**

**Supplementary Figure SFig. 1. Expression analysis of HERVFRD and EBLN family in leukemia and lymphoma cell lines and stem cell.** For each cancer entity and stem cell type, family specific FPKMs were calculated. Black dots indicate RNA-seq data from individual cell lines or stem cell samples. The bar graphs represent means and error bars indicate standard deviations. For statistical analysis, the mean values of the individual entities and stem cells were used for multiple comparisons. The dotted line represents expression in PBMCs. Raw data are available in Supplementary Table 3.

**Supplementary Table legends**

**Supplementary Table STab. 1.** Names, accession numbers and references of sequences assembled in the synthetic virus metagenome.

**Supplementary Table STab. 2.** Raw data of the FPKM values of EBV, HHV8 and XMRV in PBMCs, hematopoietic and embryonic stem cells and in cell lines of the LL-100 panel.

**Supplementary Table STab. 3.** Raw count matrix of mapped fragments, calculated family specific FPKM values, and values normalised to housekeeping genes in PBMCs, hematopoietic and embryonic stem cells and in cell lines of the LL-100 panel.

**Supplementary File legends**

**Supplementary File Viruses21.fasta.** Sequence of the synthetic virus metagenome Viruses21.

**Supplementary File Viruses21.gtf.** Annotation of the synthetic virus metagenome Viruses21.
