# Supplementary figures and images for: Identification of Differentially Expressed Human Endogenous Retrovirus Families in Human Leukemia and Lymphoma Cell Lines and Stem Cells
Source: Front Oncol. 2021 Apr 29;11:637981. doi: 10.3389/fonc.2021.637981 (PMC8117144; doi:10.3389/fonc.2021.637981)

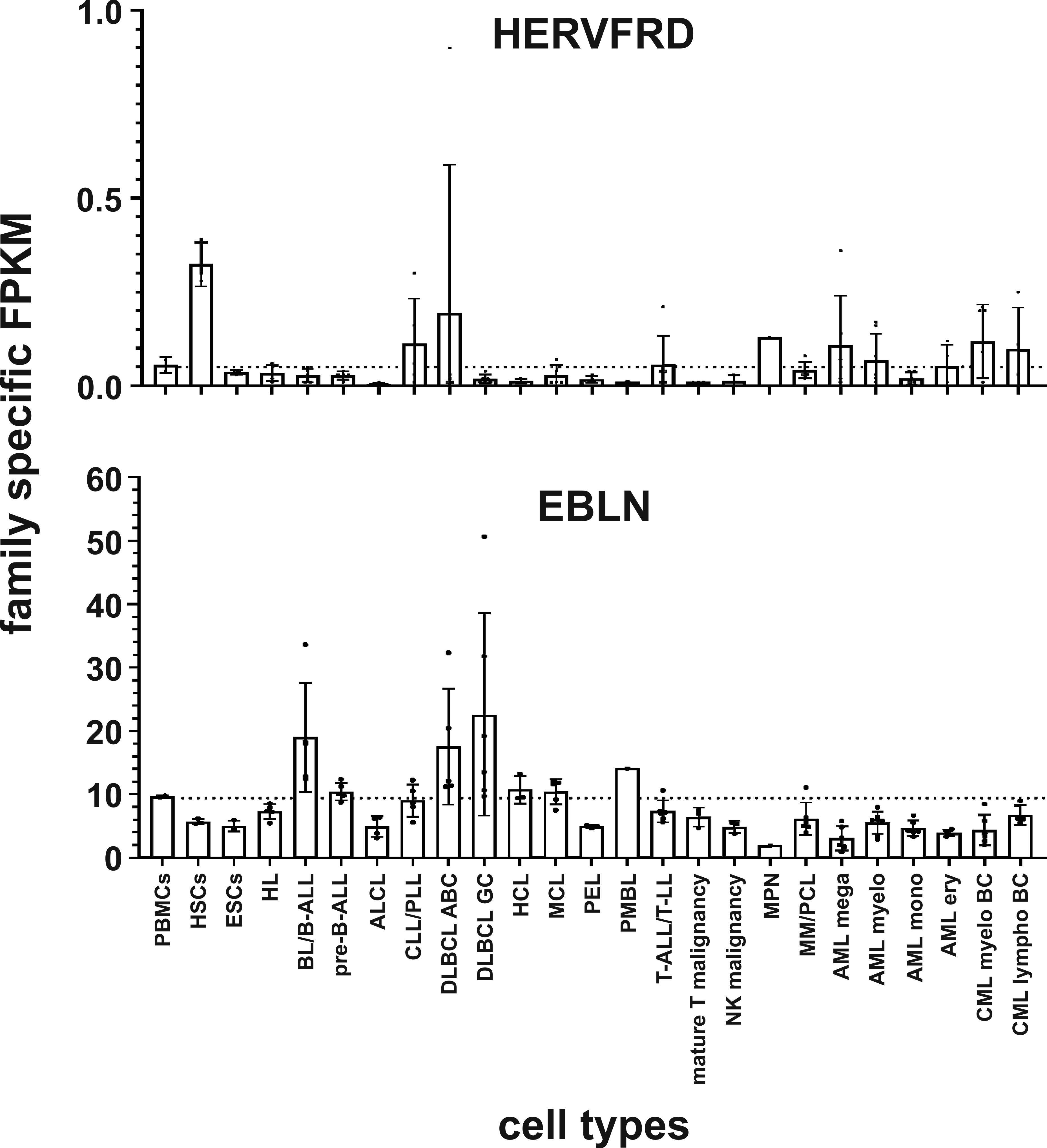

Supplement: Supplementary file 1 [file DataSheet_1.zip › 637981_Data_Sheet_1/Supplementary Figure SFig. 1.tif]
